# Integrative transcriptomic and metabolomic analyses provide insights into the effects of overexpression and knockout of *NtLHT1* in different tissues

**DOI:** 10.3389/fpls.2026.1663088

**Published:** 2026-02-06

**Authors:** Jiaxin Xing, Wenyuan Wang, Qili Mi, Lumin Zhang, Changxin Cheng, Wenwu Yang, Li Xu, Jiarui Jiang, Haiying Xiang, Wanli Zeng, Wei He, Qian Gao

**Affiliations:** 1 Technology Center, China Tobacco Yunnan Industrial Co. Ltd., Kunming, China; 2 Biotechnology and Germplasm Resources Institute, Yunnan Academy of Agricultural Sciences/Yunnan Provincial Key Lab of Agricultural Biotechnology/Key Lab of Southwestern Crop Gene Resources and Germplasm Innovation, Ministry of Agriculture and Rural Affairs, Kunming, China; 3 Honghe Prefecture Branch of Yunnan Tobacco Company, Mile, China; 4 Hongyun honghe Tobacco Group Co. Ltd., Kunming, China; 5 Technology Center, China Tobacco Fujian Industrial Co. Ltd., Xiamen, China

**Keywords:** amino acid transporter, metabolomics, NtLHT1, plant development, transcriptomics

## Abstract

Lysine-Histidine Transporter 1 (LHT1) is a key amino acid transporter involved in plant nutrition, metabolism, leaf development, and abiotic stress tolerance. However, the regulatory mechanisms of NtLHT1 (LHT1 in *Nicotiana tobacum*) across different plant organs remain poorly understood. In this study, we utilized *NtLHT1* knockout (LHT1-KO), overexpression (LHT1-OE), and wild-type (LHT1-WT) tobacco lines (cv. Honghuadajinyuan) to investigate the organ-specific effects of altered *NtLHT1* expression on metabolic and transcriptomic profiles. At the 10-leaf stage, leaves, stems, and roots were collected for transcriptome sequencing (RNA-seq) and ultra-performance liquid chromatography–tandem mass spectrometry (UPLC–MS/MS)-based metabolomic analysis, with qRT-PCR performed to validate RNA-seq results. Principal component analysis (PCA) revealed that organ identity exerted a stronger regulatory influence on the metabolome and transcriptome than *NtLHT1* genetic manipulation. LHT1-OE induced more profound metabolic and transcriptomic perturbations across organs compared to LHT1-KO: in roots, LHT1-OE upregulated pathways related to alkaloid biosynthesis and zeatin biosynthesis; in stems, it enhanced phenylpropanoid and terpenoid metabolic flux; in leaves, it repressed flavonoid and terpenoid biosynthesis by downregulating key structural genes (e.g., *ACCT*, *FPPS, TPS*) and modulated hormone homeostasis by increasing cytokinin (CTK) accumulation and decreasing auxin (IAA) and gibberellin (GA) levels. Collectively, our multi-omics analysis demonstrates that NtLHT1 acts as a “metabolic node” integrating primary and secondary metabolism as well as hormone signaling, exerting organ-specific regulatory effects on tobacco metabolism and gene expression. These findings provide a molecular framework for understanding NtLHT1’s multifunctional roles and offer potential targets for improving agronomic traits (e.g., leaf size, stress tolerance) in tobacco and other crops through genetic manipulation.

## 1 Introduction

Amino acid transporters, particularly the Lysine-Histidine Transporter 1 (LHT1), play indispensable roles in plant nutrition and metabolism by facilitating amino acids uptake and distribution across plant tissues. As a plasma membrane-localized transporter belonging to the Amino Acid-Polyamine-Choline (APC) family, LHT1 facilitates the transport of multiple amino acids, including lysine, histidine, glutamic acid, alanine, serine, proline and glycine (Svennerstam et al., 2007). First identified and functionally characterized in *Arabidopsis thaliana* (Svennerstam et al., 2008), LHT1 homologs have subsequently been investigated in economically important crops such as rice (*Oryza sativa*), maize (*Zea mays*), soybean (*Glycine max*) and tobacco (*Nicotiana tobacum*) (Wang et al., 2019; Xing et al., 2024). Although NtLHT1 (the LHT1 ortholog in *Nicotiana tobacum*) has been established as a key amino acid transporter and reported to regulate leaf morphological development as well as plant tolerance to abiotic stress, its regulatory mechanisms in different plant organs remain poorly understood (Xing et al., 2024).

Previous studies have delineated diverse physiological functions of LHT1 in plants. LHT1 is widely expressed in various tissues and is critically involved in amino acids uptake by root epidermal and cortical cells (Zhao et al., 2012). Additionally, LHT1 mediates the loading of amino acids from root symplast into xylem vessels, thereby enabling their translocation to aerial shoot tissues (Tegeder, 2012). Within leaf tissues, LHT1 is localizes to the plasma membrane of phloem companion cells, where it facilitates bidirectional amino acid transport (both efflux and influx) between the apoplast and symplasm (Guo et al., 2021). This bidirectional transport capability allowsLHT1 to efficiently allocate amino acids to both source tissues (e.g., mature leaves) and sink tissues (e.g., developing organs). Furthermore,LHT1 expressed in leaf vascular tissues participates in the long-distance translocation of amino acids to seeds and heterotrophic organs (Luo et al., 2018; Guo et al., 2020). beyond its roles in plant nutrition, LHT1 has also been implicated in leaf defense responses through the regulation of amino acid-derived secondary metabolites (Hirner et al., 2006). Collectively, these findings highlight the tissue-specific functions of LHT1 in mediating cellular amino acid uptake, vascular transport, source-sink allocation, and defense-related metabolism.

Despite significant progress in elucidating the molecular characteristics and physiological roles of LHT1, the effects of modulating LHT1 expression on plant metabolic networks and organ-specific gene expression profiles remain insufficiently understood. In the present study, we integrated metabolite profiling and transcriptome analysis to investigate *NtLHT1* knockout and overexpression tobacco lines, focusing on three key plant organs: leaves, stems, and roots. Our multi-omics dataset provides novel insights into the heterogeneous, tissue-specific impacts of altered *NtLHT1* expression on plant metabolic pathways. The organ-specific metabolic perturbations observed in this study further underscore the complexity of NtLHT1 functionality across distinct biological contexts.

## 2 Materials and methods

### 2.1 Plant materials and sampling

The F_2_ homozygous and T-DNA-free seeds of *NtLHT1* (*Ntab0818090*) knockout mutants (LHT1-KO) used in this study were generated via the CRISPR/Cas9 gene editing system (Zhang et al., 2023). The F_2_ tobacco plants overexpressing *NtLHT1* (LHT1-OE) used in this study were previously generated and validated (Xing et al., 2024). Both lines were developed in the background of tobacco (cv. Honghuadajinyuan), designated as LHT1-WT (wild type). Quantitative real-time polymerase chain reaction (qRT-PCR) results showed that the *NtLHT1* was highly overexpressed in LHT1-OE plants and barely expressed in LHT1-KO plants compared to LHT1-WT (Xing et al., 2024).

Seeds of LHT1-WT, LHT1-KO, and LHT1-OE were directly germinated in seedling trays for 45 days. Seedlings were then transplanted into pots and cultured in a phytotron under conditions of 16-h light/8-h dark (25 °C) and 70% relative humidity for 25 days. At the 10-leaf stage, the 3rd and 4th leaves from the top, stems and roots were collected from LHT1-WT, LHT1-KO, and LHT1-OE plants comprised of eight biological replicates. Four replicates were taken for transcriptome and others were used for the non-targeted metabolomics. The samples included the leaves, roots, and stems of wild-type cv. Honghuadajinyuan seedlings (HSL, HSR and HSS), of *NtLHT1* knockout seedlings (LKSL, LKSR and LKSS), and of *NtLHT1* overexpression seedlings (LOSL, LOSR and LOSS). These samples were snap-frozen in liquid nitrogen and stored at -80 °C for subsequent experiments.

### 2.2 RNA extraction, cDNA library construction and transcriptome sequencing

Total RNA was extracted from each sample using the FastPure Plant Total RNA Isolation Kit (Vazyme, Nanjing, China) according to the manufacturer’s protocol. A total of 36 samples from four biological replicates were collected. RNA integrity and contamination were assessed by 1% agarose gel electrophoresis, and RNA purity was evaluated with a Nanophotometer spectrophotometer (IMPLEN, CA, USA). After ensuring RNA quality, mRNA was enriched using magnetic beads with Oligo(dT) tails. The first strand of cDNA is then synthesized using a six-base random primer, and the second strand of cDNA is synthesized by adding buffer, dNTPs, RNase H, and DNApolymerase I (NEB, Massachusetts, USA). Library preparation and sequencing were performed by BGI Genomics Co., Ltd. (Wuhan, China).Transcriptome Analysis.

Raw RNA-seq data were quality-controlled using the DNBSEQ Sequencing Platform, involving the removal of low-quality reads, adaptor sequences, and reads with high levels of N bases. Read alignment to the reference genome sequence (https://solgenomics.net/ftp/genomes/Nicotiana_tabacum/edwards_et_al_2017/assembly/) was performed with HISAT2 using default parameters. Reads were further filtered with *SAMTOOLS* (v 1.9; https://github.com/samtools/samtools) using a Q20 threshold. Gene expression levels were quantified by counting reads mapped to each gene with FeatureCounts v1.5.0, and fragments per kilobase of exon model per million mapped fragments (FPKM) values were calculated based on gene length and read count. Differentially expressed genes (DEGs) were identified using DESeq2 (Love et al., 2014) with the criteria of |log_2_ (fold change)| > 0.75 and p-value < 0.05. Statistics on differentially expressed genes between different tissues were derived. Correlation analyses of the DEGs were conducted using R software. KEGG pathway enrichment analysis of DEGs was performed with the phyper modules from R software, and P values were corrected using the false discovery rate (FDR) method. A Q value ≤ 0.05 was considered as significant enrichment, where the Q value is the adjusted P value.

### 2.3 qRT-PCR validation of RNA-seq results

To validate the accuracy of differential gene expression patterns related to plant hormone, 6 genes in leaves were selected for qRT-PCR verification. Specific primers were designed using Primer-BLAST (https://www.ncbi.nlm.nih.gov/tools/primer-blast/). cDNA was synthesized from 1 mg of total RNA using the HiScript III RT SuperMix kit (Vazyme, Nanjing, China) according to the manufacturer’s instructions. Quantitative Real-time PCR (qRT-PCR) was performed using the ChamQ Universal SYBR qPCR Master Mix (Vazyme) on a LightCycler 96 Real-time PCR system (Roche), with the tobacco NtEF1 as the internal control (Schmidt and Delaney, 2010). The PCR conditions were 95 °C for 30 sec, followed by 40 cycles of 95 °C for 5 sec and 60 °C for 30 sec. The relative gene expression levels of the interest genes were quantified using the 2^−ΔΔCt^ method as previously described (Livak and Schmittgen, 2001). Each sample was analyzed with three biological replicates and technical replicates. And the primer sequences used are provided in **Supplementary Table S1**.

### 2.4 Metabolites extraction

Metabolomic analysis was performed by Wuhan Metware Biotechnology Co., Ltd (http://www.metware.cn/) with four biological replicates. Samples were freeze-dried and ground intopowder using a mixer mill (MM 400, Retsch) with zirconium beads at 30 Hz for 1.5 min. Approximately 100 mg of powder was weighed and extracted overnight at 4 °C in 1.2 mL of 70% (v/v) aqueous methanol. After centrifugation at 12,000×g for 10 min, the filtered extracts were preserved at -80°C for subsequent ultra-performance liquid chromatography–tandem mass spectrometry (UPLC–MS/MS) analysis.

### 2.5 UPLC–MS/MS analysis

Metabolic profiling of the samples was conducted utilizing an LC-QTOF/MS system, which included a Shimadzu LC-20A liquid chromatography system (HPLC) and a Sciex 5600 triple TOF MS/MS system (QTOF/MS) equipped with an electrospray DuoSpray ion source. The system was operated in both positive and negative ionization modes. For positive ionization, the mobile phase comprised 0.1% formic acid in water (A) and 0.1% formic acid in acetonitrile (B), while for negative ionization, it consisted of 5 mM ammonium acetate in water (A) and acetonitrile (B). Gradient elution was employed, with a mass scan range set between 50 and1000 m/z. The data acquired were analyzed using full-scan mode in conjunction with information-dependent analysis (IDA). Source voltages, gas pressures, collision energies, and other parameters were set accordingly for each ionization mode.

### 2.6 Metabolomic data preprocessing and annotation

The raw data obtained from LC-QTOF/MS were processed using the MS-DIAL software (v4.90) for peak collection, baseline filtering and calibration, peak alignment, deconvolution, and peak area integration. Compound identification was performed by matching retention time (RT± 0.2), mass accuracy (<10 ppm), isotope ratio, and MS/MS spectra to public databases including METLIN, HMDB, and Metware in-house metabolite database. Multivariate data analysis was conducted using MATLAB (R2021b), including principal component analysis (PCA) to assess overall sample variation and partial least-squares discriminant analysis (PLS-DA) to identify metabolites contributing to group differences. Significant differences metabolites (DMs) were determined based on two criteria: variable importance in projection (VIP) values>1 (from PLS-DA models) and t-tests with p < 0.05 (95% confidence level). MS-FINDER software (v4.0) was used for metabolite structure elucidation, and the KEGG pathway enrichment analysis of DMs was performed using the KEGG database (https://www.genome.jp/kegg/) with p < 0.05 as the significance threshold.

## 3 Results

### 3.1 Disturbance of total metabolite content in various organs by NtLHT1 knockout and overexpression

**Figure 1:**
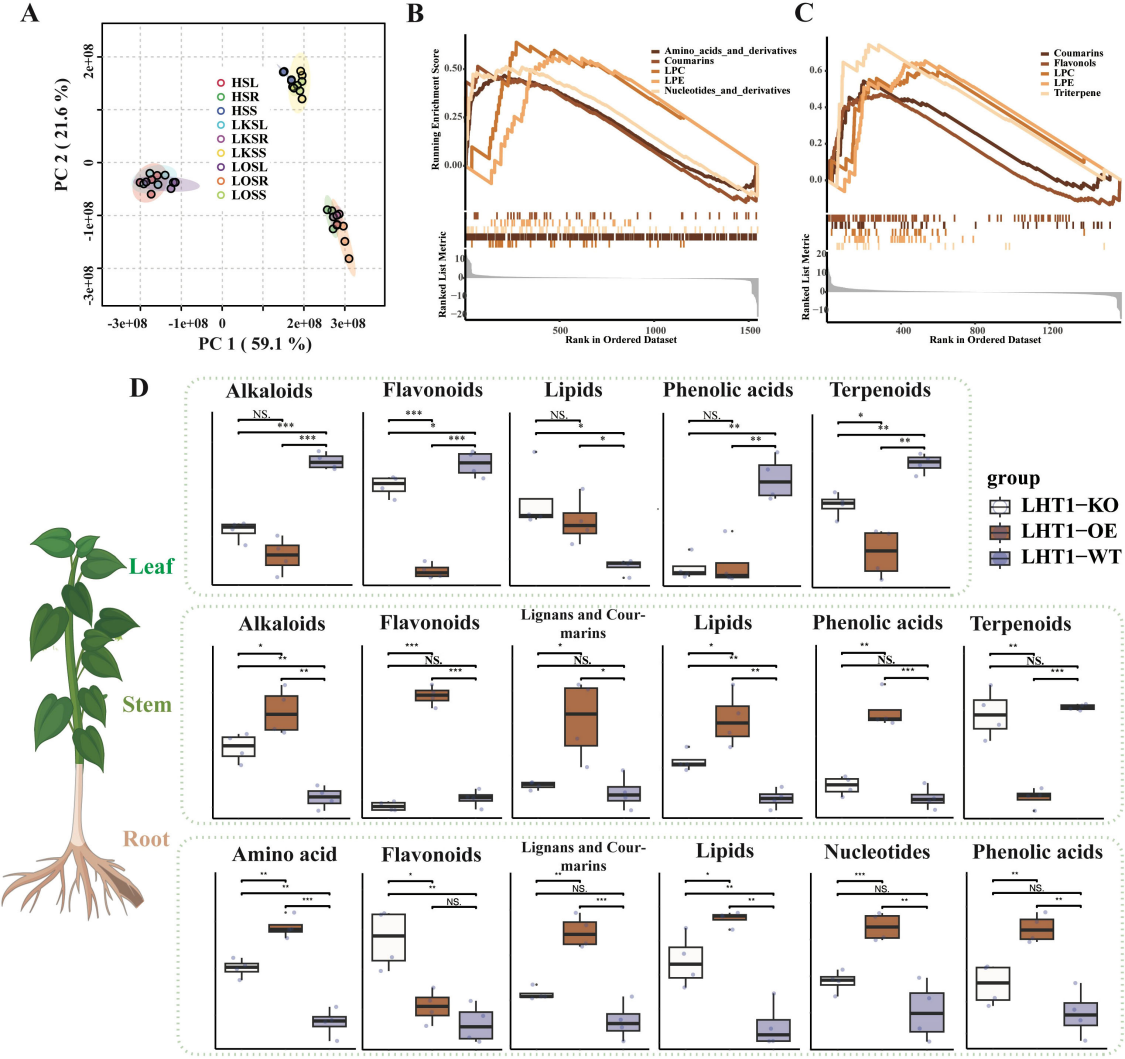
Effects of NtLHT1 overexpression and knockout on the metabolite composition in tobacco root, stem, and leaf tissues. **(A)** PCA analysis based on metabolomic data of tobacco root, stem, and leaf samples. PC1 and PC2 axes represent different directions of metabolic changes. *HSL*, *HSR* and *HSS*, LHT1-WT seedling leaf, root and stem, *LKSL*, *LKSR* and *LKSS*, LHT1-KO seedling leaf, root and stem, *LOSL*, *LOSR* and *LOSS*, LHT1-OE seedling leaf, root and stem. **(B)** and **(C)** Enrichment analysis of metabolites affected by LHT1-OE in the root and stem. The x-axis represents the position of metabolites in the ranked list of differentially regulated metabolites, and the y-axis represents the enrichment results. The upward curve indicates significant enrichment. **(D)** Quantitative effects of NtLHT1 overexpression and knockout on major metabolic categories in the root, stem, and leaf. *P < 0.05, **P < 0.01, ***P < 0.001 mean values were significantly difference by two-tailed Student’s *t* test.

Metabolomic profiling was performed on roots, stems, and leaves of tobacco lines with wild-type NtLHT1 (LHT1-WT), NtLHT1 knockout (LHT1-KO), and NtLHT1 overexpression (LHT1-OE), with raw data provided in **Supplementary Table S2**. Principal component analysis (PCA) revealed a more prominent separation among different organs than among the three genotypes (**Figure 1A**). Specifically, PC1 effectively distinguished leaves from roots and stems, consistent with the unique photosynthetic metabolic specialization of leaves; PC2 further resolved metabolic differences between roots and stems, likely reflecting tissue-specific roles in nutrient transport and secondary metabolism. This hierarchical separation indicates that organ identity exerts a stronger regulatory influence on the metabolome than NtLHT1 genetic manipulation.

To characterize genotype-driven metabolic shifts, metabolite class enrichment analysis was conducted based on differential metabolites identified in each organ. For LHT1-OE lines: in roots, significant accumulation was observed for amino acids and their derivatives, coumarins, lysophosphatidylcholines (LPC), lysophosphatidylethanolamines (LPE), and nucleotides and their derivatives (**Figure 1B**); in stems, enrichment was detected for coumarins, flavonols, LPC, LPE, and triterpenoids (**Figure 1C**), suggesting the enhanced metabolic flux through the phenylpropanoid and terpenoid pathways; in leaves, coumarins, triterpenoids, vitamins, and LPE were significantly enriched (**Supplementary Figure S1A**), suggesting a redirection of carbon resources toward lipid and antioxidant biosynthesis. In contrast, LHT1-KO induced only modest metabolic perturbations across all organs (**Supplementary Figure S2**). The magnitude of metabolic changes in LHT1-KO lines was substantially smaller than that in LHT1-OE lines, with key affected metabolite classes limited to amino acids and their derivatives, LPC, LPE, flavones, flavonols, and alkaloids. This indicates that NtLHT1 knockout disrupts basal amino acid transport homeostasis but does not alter the global metabolic architecture of tobacco organs.

Quantitative statistical analysis was further conducted to quantify changes in total metabolite abundance across LHT1-WT, LHT1-KO, and LHT1-OE lines (**Figure 1D**). In leaves, LHT1-OE and LHT1-KO exhibited a conserved metabolic metabolic trend: alkaloids, flavonoids, phenolic acids, and terpenoids were significantly reduced, while lipids were significantly increased. In stems, genotype-specific differences emerged: LHT1-OE drove extensive accumulation of major metabolites (alkaloids, flavonoids, lignans and coumarins, lipids and phenolic acids) except for a reduction in terpenoids; LHT1-KO, however, only induced increase in alkaloids and lipids. In roots, LHT1-OE triggered synergistic upregulation of amino acids, lignans and coumarins, lipids, nucleotides, and phenolic acids; in contrast, LHT1-KO roots showed only modest increases in amino acids and flavonoids, with no significant changes in other metabolite classes.

Collectively, these results demonstrate that NtLHT1 functions as a key regulator of organ-specific metabolism in tobacco: overexpression amplifies biosynthetic capacity for nitrogen-containing metabolites (e.g., amino acids, nucleotides) and secondary metabolites (e.g., coumarins, flavonols) in a tissue-dependent manner, while knockout primarily disrupts amino acid homeostasis with limited compensatory metabolic shifts.

### 3.2 Differences and functional changes of metabolites in different organs induced by NtLHT1 knockout and overexpression

**Figure 2:**
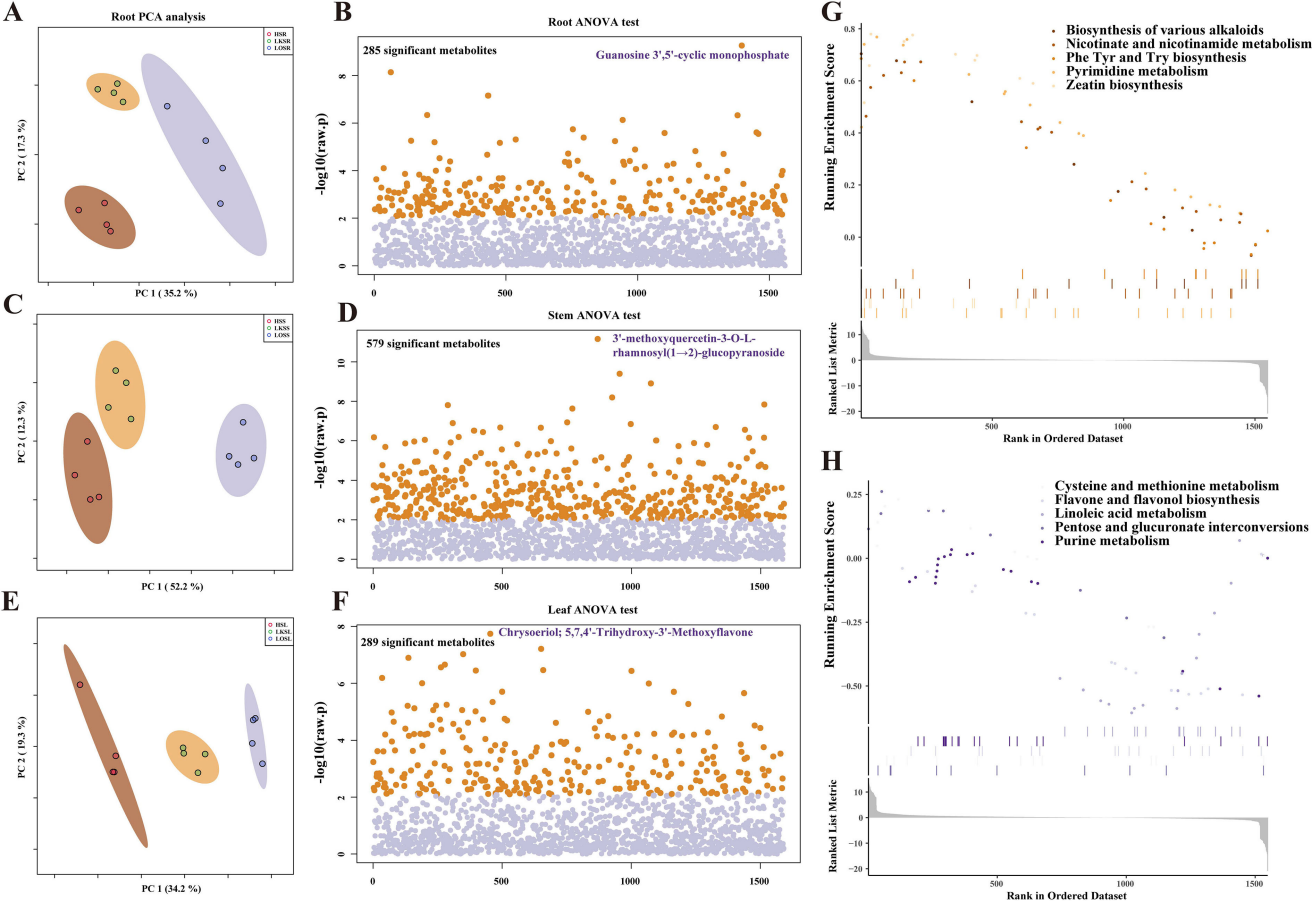
Identification and functional analysis of differentially regulated metabolites in tobacco root, stem, and leaf tissues induced by NtLHT1 overexpression and knockout. **(A, C, E)** Distribution of samples in the PCA analysis of root, stem, and leaf. **(B, D, F)** Screening of differentially regulated metabolites among LHT1-WT, LHT1-OE, and LHT1-KO in root, stem, and leaf tissues based on ANOVA. The y-axis represents the logarithm of p-values, and the x-axis represents the position of metabolites in the ranked list of differential regulation. **(G, H)** Pathway enrichment analysis of up and down regulated metabolites in the root of LHT1-OE compared with LHT1-KO.

NtLHT1 overexpression (OE) and knockout (KO) triggered significant alterations in the metabolite profiles of tobacco, which were systematically characterized in this study. Analysis of variance (ANOVA) was applied to quantify the specific metabolite changes induced by LHT1-OE/KO across different plant organs. Principal component analysis (PCA) demonstrated that in the roots and leaves, the first two principal components (PC1 and PC2) prominently separated wild-type (LHT1-WT) samples from the LHT1-OE/KO counterparts (**Figures 2A, E**). In contrast, in stems, PC1 and PC2 primarily distinguished LHT1-OE samples from the other two genotypes (**Figure 2C**).

Following ANOVA analysis, 285, 579, and 289 differential metabolites (DIMs) were detected among LHT1-WT, LHT1-OE and LHT1-KOgenotypes in roots, stems, and leaves, respectively (**Figures 2B, D, F**). Additional ANOVA was conducted on LHT1- WT/OE/KO plants across distinct organs (**Supplementary Table S3**, **S4** and **S5**), and the results indicated that metabolite differences between organs were substantially greater than those induced by LHT1-OE/KO (**Supplementary Figure S3**).

Further functional enrichment analysis revealed that LHT1-OE upregulated metabolic pathways in roots, predominantly involving Biosynthesis of various alkaloids, zeatin biosynthesis, phenylalanine (Phe), tyrosine (Tyr) and tryptophan (Trp) biosynthesis, as well as nicotinate and nicotinamide metabolism(**Figure 2G**). conversely, the downregulated pathways included purine metabolism, pentose and glucuronate interconversions, linoleic acid metabolism, lavone and flavonol biosynthesis, and cysteine and methionine metabolism (**Figure 2H**). In leaves, LHT1-OE significantly upregulated pathways such as arginine biosynthesis, ascorbate and aldarate metabolism, cysteine and methionine metabolism, lysine degradation, and zeatin biosynthesis. The downregulated pathways, however, included tyrosine metabolism, tryptophan metabolism, galactose metabolism, flavone and flavonol biosynthesis, and biosynthesis of various alkaloids (**Supplementary Figure S4A, B**). In stems, LHT1-OE induced upregulated pathways encompassed ABC transporters, ascorbate and aldarate metabolism, cysteine and methionine metabolism, purine metabolism, and zeatin biosynthesis. The downregulated pathways included tryptophan metabolism, phenylpropanoid biosynthesis, phenylalanine tyrosine and tryptophan biosynthesis, flavonoid biosynthesis, as well as flavone and flavonol biosynthesis (**Supplementary Figure S4C, D**).

Collectively, these findings suggest that cysteine and methionine metabolism, zeatin biosynthesis, flavone and flavonol biosynthesis, and aromatic amino acids (Phe/Tyr/Trp) biosynthesis are the core metabolic pathways perturbed by LHT1-OE/KO.

### 3.3 Dynamic transcriptomic changes in various organs induced by NtLHT1 knockout and overexpression

**Figure 3:**
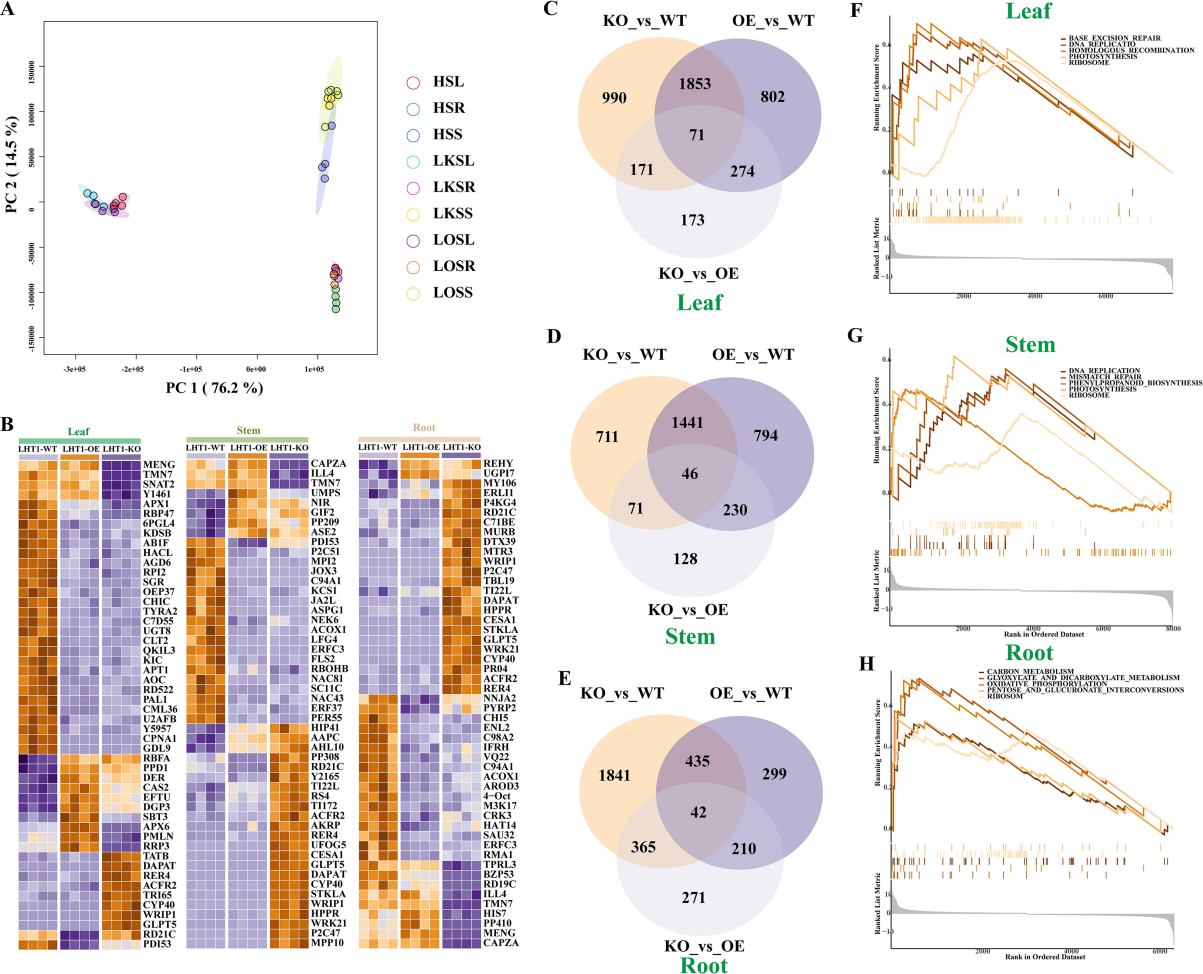
Effects of NtLHT1 overexpression and knockout on the transcriptome of tobacco root, stem, and leaf. **(A)** PCA analysis based on transcriptomic data of tobacco root, stem, and leaf samples. **(B)** Heatmap displaying a subset of 50 differentially expressed genes affected by LHT1-OE and LHT1-KO in roots, stems, and leaves. Blue represents downregulation, while red represents upregulation. **(C–E)** Comparison of the number of differentially expressed genes among LHT1-WT, LHT1-OE, and LHT1-KO groups in the leaf, stem, and root. **(F–H)** Pathway enrichment analysis of differentially expressed genes induced by LHT1-OE in the leaf, stem and root.

To elucidate the molecular mechanisms underlying the metabolite alterations induced by LHT1-OE/KO, RNA sequencing (RNA-seq)-based transcriptomic analysis was performed on all samples. PCA of transcriptomic data indicated that the degree of separation between different organs was significantly greater than that between LHT1-WT, LHT1-KO, and LHT1-OE plants (**Figure 3A**), which was consistent with the metabolomic PCA results, reflecting the coordinated transcriptomic and metabolic responses to organ identity. Specifically, PC1 effectively distinguished leaves from roots and stems, while PC2 clearly separated all three organs from each other. Pairwise differential expression analysis identified organ-specific differentially expressed genes (DEGs) between genotypes: in leaves, 802 and 990 DEGs were identified in the LHT1-OE and LHT1-KO groups, respectively (**Figure 3C**), with LHT1-OE DEGs primarily involved in base excision repair, DNA replication, photosynthesis, and ribosomes (**Figure 3F**). In stems, 794 and 711 DEGs were identified in LHT1-OE and LHT1-KO groups (**Figure 3D**), with LHT1-OEDEGs chiefly participating in phenylpropanoid biosynthesis, DNA replication, photosynthesis, and ribosomes (**Figure 3G**). In roots, the number of DEGs was notably asymmetric between genotypes: 299 in LHT1-OE and 1841 in LHT1-KO (**Figure 3E**), with the LHT1-OE DEGs enriched in carbon metabolism, oxidative phosphorylation, pentose and glucuronate interconversions, glyoxylate and dicarboxylate metabolism (**Figure 3H**).

A cross-organ analysis further screened 50 core genes consistently regulated by LHT1-OE/LHT1-KO across roots, stems, and leaves (**Figure 3B**). In leaves, both LHT1-OE and LHT1-KO induced more downregulated than upregulated DEGs. Specifically, *MENG*, *TMN7*, *Y1461*, and *SNAT2* (involved in photosystem maintenance) were leaf-specific DEGs downregulated in LHT1-KO, while *RBFA*, *PPD1*, *DER*, and *APX6* (related to ribosome assembly and oxidative stress) were upregulated in LHT1-OE. In stems, *UMPS*, *NIR*, and *GIF2* (associated with nitrogen metabolism and cell growth) were significantly upregulated in LHT1-OE, whereas in roots, LHT1-OE specifically induced the upregulation of *REHY* (glyoxylate cycle) and *UGP17* (glycosylation). These results demonstrated that NtLHT1 overexpression or knockout elicits organ-specific transcriptomic changes in tobacco. At the transcriptomic level, phenylpropanoid biosynthesis, DNA replication, photosynthesis, carbon metabolism and oxidative phosphorylation represent the most significantly altered pathways, which are partially overlapping with the core metabolic pathways identified in metabolomic analysis.

### 3.4 NtLHT1 overexpression represses terpenoid metabolism and biosynthesis in leaves

**Figure 4:**
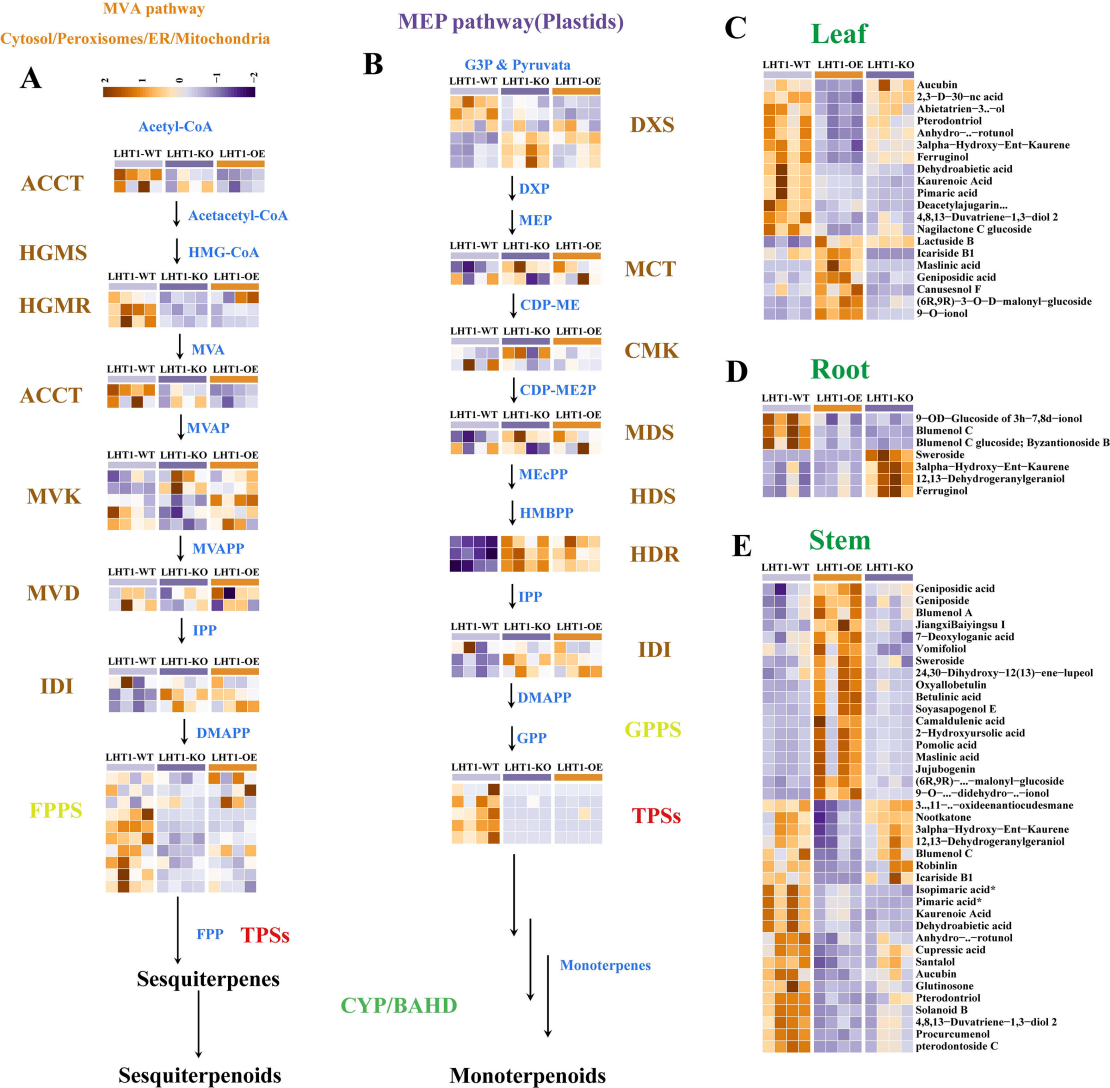
Effects of NtLHT1 overexpression and knockout on tobacco terpene metabolism. **(A, B)** Schematic representation of terpene biosynthesis MEP and MVA pathways. **(C–E)** Relative levels of selected terpenes among LHT1-WT, LHT1-OE, and LHT1-KO in tobacco leaves, roots and stems.

Metabolomic analysis revealed that both LHT1-OE and LHT1-KO led to a significant reduction in total terpenoid content in tobacco leaves. Targeted analysis of terpenoid profiles across organs (**Figures 4C-E**) showed that in leaves, most of the 20 significant altered metabolites, including 3α-hydroxy-ent-kaurene, kaurenoic acid, and pterodontriol, all of which were downregulated in LHT1-OE/KO plants (**Figure 4C**).

To dissect the regulatory mechanism underlying terpene reduction, we systematically identified structural genes involved in terpene biosynthesis and reconstructed the pathway in tobacco leaves (**Figures 4A, B**). Terpenoid biosynthesis in tobacco relies on two conserved pathways: mevalonic acid (MVA) pathway and the methylerythritol phosphate (MEP) pathway. A total of 11 key structural genes spanning both pathways were identified, including *ACCT*, *HGMR*, *MVK*, *MVD*, *IDI*, *FPPS*/*GPPS*, *DXS*, *MCT*, *CMK*, *MDS*, *HDR*, and *TPS*. Expression analysis of these genes in leaves showed that *ACCT*, *HGMR*, *FPPS* and *TPS* were downregulated in both LHT1-OE and LHT1-KO plants. Notably, ACCT (rate-limiting enzyme of MVA pathway) and TPS (terpene synthase) are core regulators of plant terpenoid biosynthesis. These results suggest that LHT1-OE/KO may block both MVA and MEP pathways, leading to reduced accumulation of sesquiterpenoids, diterpenoids, and monoterpenoids in tobacco leaves.

### 3.5 NtLHT1 overexpression inhibits flavonoid metabolism and biosynthesis in leaves

**Figure 5:**
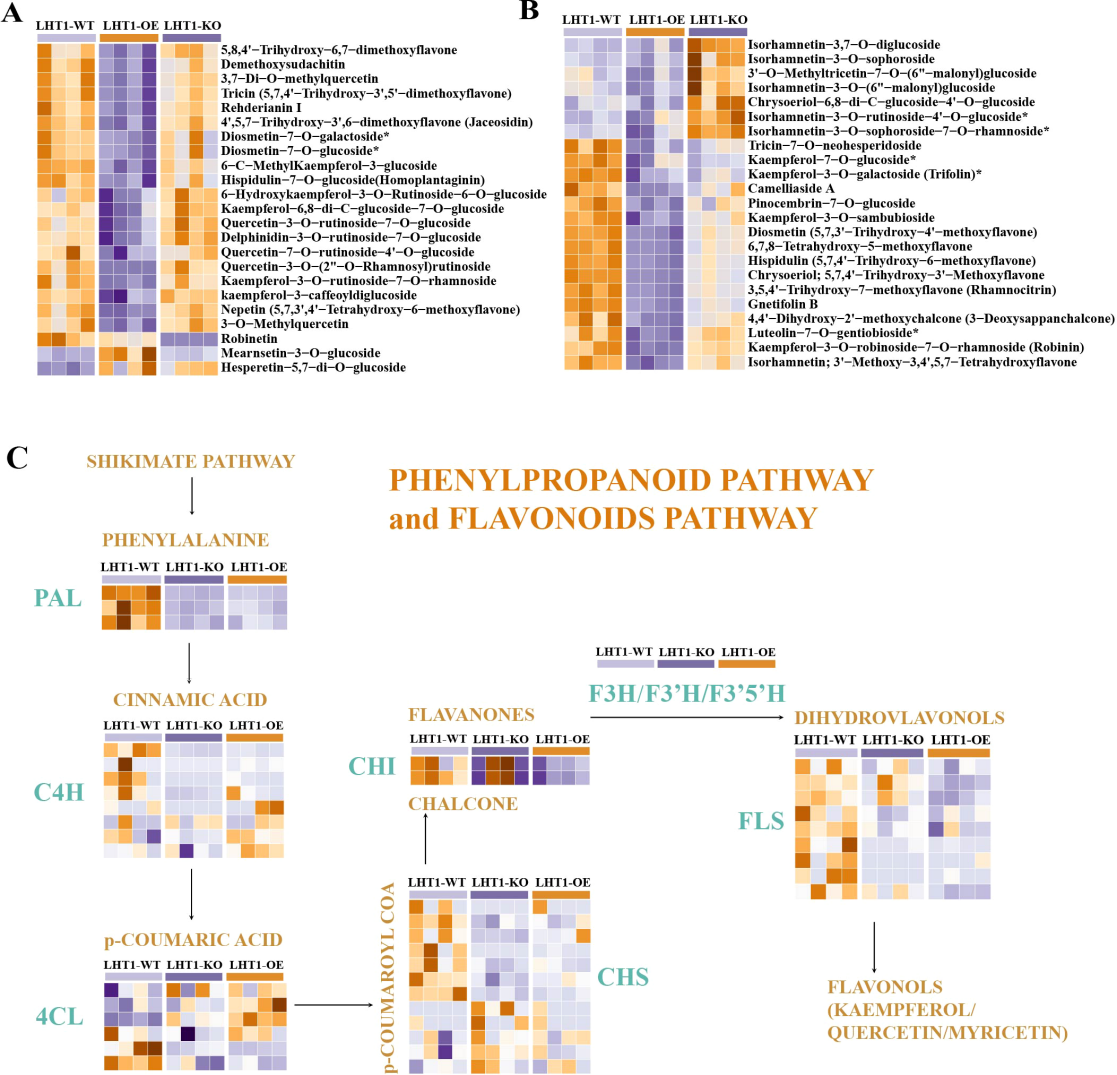
Effects of NtLHT1 overexpression and knockout on tobacco leaf flavonoid metabolism. **(A, B)** Heatmap displaying the relative levels of selected flavonoid compounds in tobacco leaves of LHT1-WT, LHT1-OE, and LHT1-KO plants. **(C)** Simplified schematic representation of flavonoid biosynthesis pathway, showing major enzymatic reactions and intermediates.

Metabolomic analysis indicated that LHT1-OE caused a profound reduction in flavonoid metabolites: among 43 differential flavonoid metabolites between LHT1-WT and LHT1-OE, 40 (93.0%) were downregulated in LHT1-OE (**Figures 5A, B**). These downregulated metabolites were predominantly flavones (e.g., Quercetin-3-O-rutinoside-7-O-glucoside, kaempferol-3-caffeoyldiglucoside, kaempferol-7-O-glucoside), suggesting that LHT1-OE represses the phenylpropanoid-flavonoid pathway in tobacco seedling leaves.

To verify this, we identified the structural genes in the flavonoid biosynthesis pathway (*PAL*, *C4H*, *4CL*, *CHS*, *CHI*, *FLS*) and analyzed their expression patterns. Among the tested genes (3 *PAL*, 8 *C4H*, 6 *4CL*, 2 *CHI*, 12 *CHS*, 9 *FLS*), majority were downregulated in LHT1-OE leaves (**Figure 5C**).

This expression pattern was highly consistent with the metabolomic data, confirming that *NtLHT1* overexpression inhibits both flavonoid metabolite accumulation and the expression of biosynthesis-related structural genes. These results align with the previously reported decrease in aromatic amino acid levels (Phe, Trp, Tyr) in LHT1-OE plants (Xing et al., 2024), as these amino acids serve as precursors for flavonoid biosynthesis.

### 3.6 NtLHT1 overexpression modulates phytohomone homeostasis by regulating IAA and cytokinin pathways in leaves

**Figure 6:**
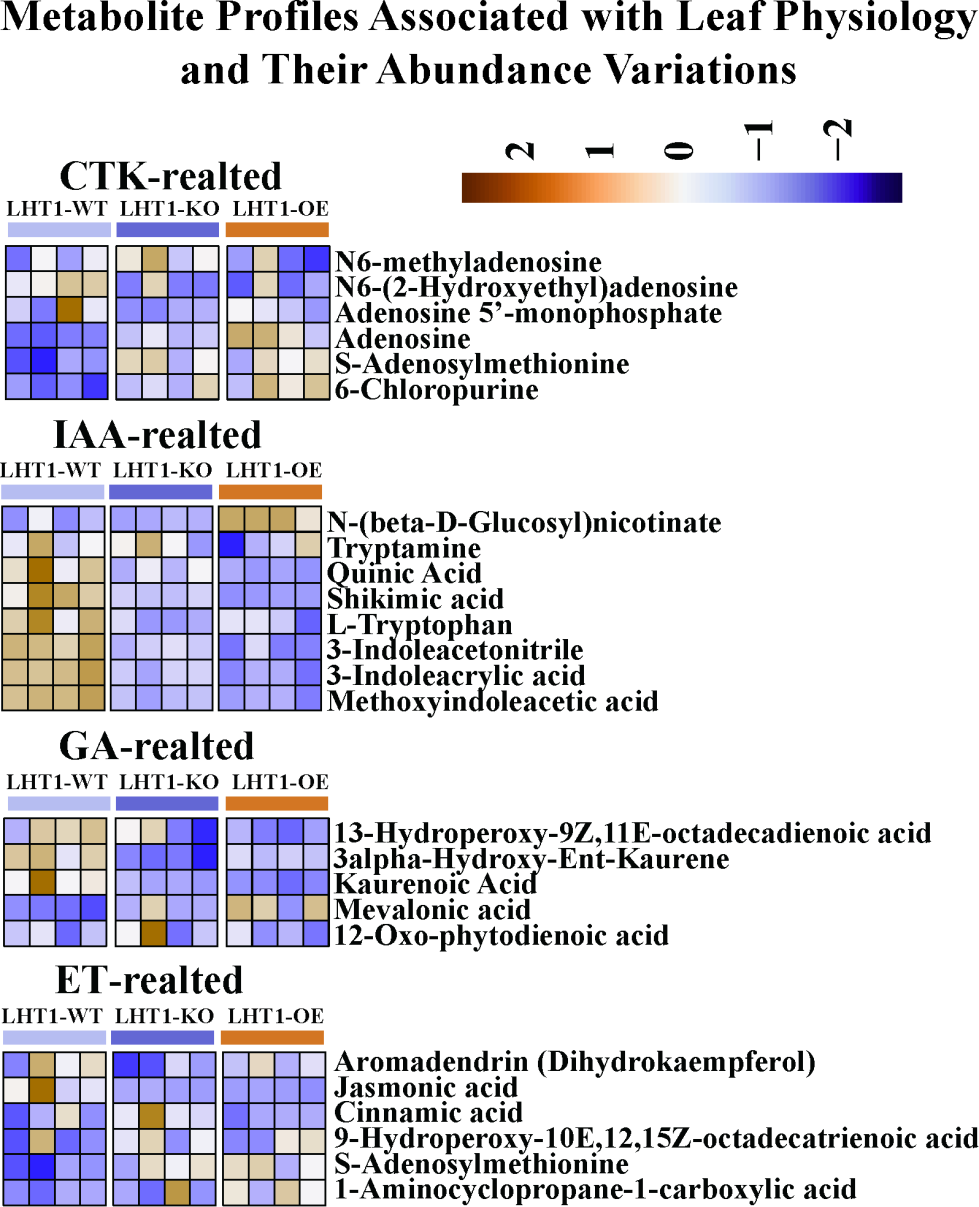
Metabolite profiles associated with leaf physiology and their abundance variations.

Plant hormones (gibberellins [Gas], cytokinins [CTKs], auxins [IAAs] and ethylene [ETH]) play vital roles in determining plant architecture traits (Ali et al., 2020). Given that LHT1-OE alters tobacco leaf morphology (Xing et al., 2024), we analyzed hormone-related metabolites and genes. Metabolomic analysis showed that LHT1-OE significantly increased CTK-related metabolites (such as Adenosine, S-Adenosylmethionine, 6-Chloropurine and Isopentenyladenine-7-N-glucoside), while decreasing IAAs and GAs in leaves. In contrast, LHT1-KO only reduced IAAs and GAs levels (**Figure 6**; **Supplementary Figure S5**).

**Figure 7:**
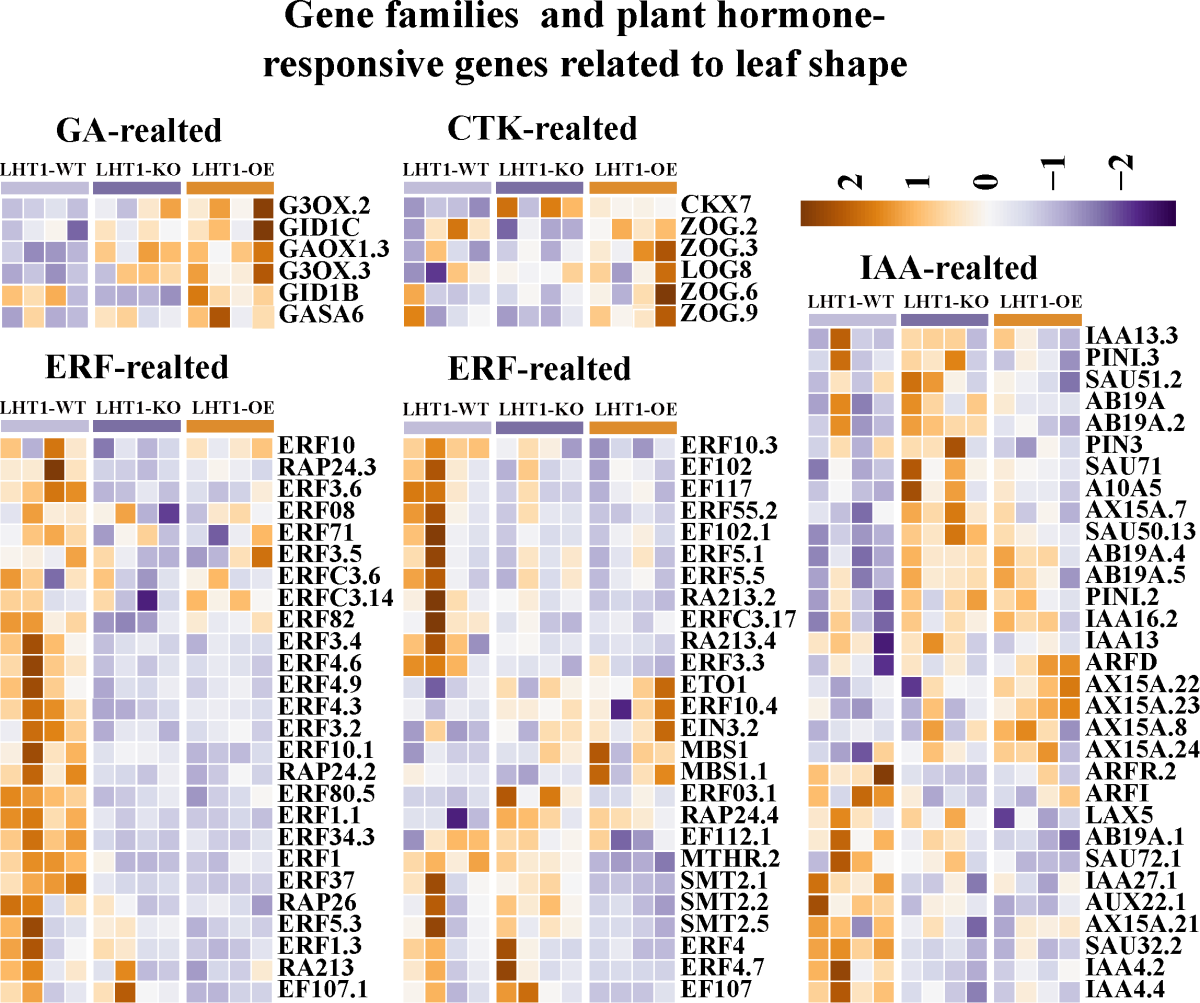
The heatmap of plant hormone responsive genes related to leaf shape. These include GA-related genes, CTK-related genes, ERF transcription factor family genes and IAA-related genes. The heatmap shows the expression changes of these gene families in LHT1-WT, LHT1-KO and LHT1-OE groups.

Transcriptomic analysis identified key hormone-related genes regulated by LHT1-OE: GA-related genes (*G3OX.2*, *GID1C*, *GAOX1*, *G3OX.3*, *GID1B*, *GASA6*) were significantly upregulated, while CTK-related genes (*CKX7*, *ZOG*, *LOG*) were specifically induced—consistent with increased CTK accumulation. Additionally, most ERF transcription factor genes (key ETH signaling components) were downregulated in both LHT1-OE/KO, whereas some IAA-related genes (*IAA13.3*, *PIN1.3*, *SAU51.2*, *AB19A*, *PIN3*) were upregulated in LHT1-KO (**Figure 7**).

Additionally, six phytohomone related DEGs were randomly selected for quantitative real-time PCR (qRT-PCR) analysis to validate the reliability of RNA-seq data. Among these, five genes (*IAA16*, *RAP26*, *ERF1*, *ERF3* and *ERF5*) were downregulated in both LHT1-OE and LHT1-KO, while *AB19A* was upregulated in LHT1-KO but downregulated in LHT1-OE. Results showed that all six genes exhibited a consistent expression pattern in both the qRT-PCR and RNA-seq analyses (**Supplementary Figure S6**).

Mechanistically, LHT1-OE reduced phenylpropanoid-derived metabolites (L-tryptophan, tryptamine, shikimic acid, quinic acid, cinnamic acid)—key IAA precursors—and increased adenosine-associated metabolites (adenosine, S-adenosylmethionine, 1-aminocyclopropane-1-carboxylic acid)—critical for CTK/ETH biosynthesis. Collectively, LHT1-OE regulates leaf development primarily by modulating phenylpropanoid and adenosine metabolic branches to rewire hormone homeostasis.

## 4 Discussion

Leaves serve as the primary photosynthetic organs of plants and play a pivotal role in determining canopy architecture, which directly modulates crop yield potential (Du et al., 2018). Leaf development is a complex process coordinated by multiple hormones, including auxin, cytokinin, and GA, which regulate leaf primordium initiation (Reinhardt et al., 2000), vascular formation (Sieburth, 1999), and cell division/expansion (Keller, 2017). Our study reveals that the amino acid transporter NtLHT1 acts as a key regulator of leaf development by integrating hormone signaling and metabolic networks in tobacco.

Cytokinin is essential for shoot apical meristem (SAM) maintenance (Besnard et al., 2014), and acts downstream of KNOX1 to sustain stem cell activity (Hay and Tsiantis, 2010). Our findings extend previous observations that NtLHT1 affects leaf morphology by demonstrating that LHT1-OE-induced leaf widening and area expansion (Xing et al., 2024) are associated with activated CTK signaling: CTK-related genes (*CKX7*, *ZOG*, *LOG*) and zeatin biosynthesis pathways were upregulated, leading to increased CTK accumulation. Additionally, LHT1-OE upregulated GA-related genes (*G3OX*, *GID1C*, *GASA6*), suggesting a coordinated regulation of CTK and GA pathways—consistent with their synergistic role in leaf growth (Wu et al., 2021; Ritonga et al., 2023). These results indicate that NtLHT1 modulates leaf development by rewiring CTK, GA, and IAA signaling networks.

Flavonoids are multifunctional metabolites involved in antioxidant defense, UV protection, and stress resistance (Ramaroson et al., 2022). Our study shows that LHT1-OE significantly inhibits flavonoid accumulation by downregulating key biosynthesis genes (*PAL*, *C4H*, *CHS*). This inhibition is likely linked to reduced aromatic amino acid precursors (Phe, Trp, Tyr) in LHT1-OE plants as these amino acids are the starting point of the phenylpropanoid pathway. Given the role of flavonoids in stress responses, NtLHT1 may mediate plant stress tolerance by regulating flavonoid metabolism—an avenue requiring further investigation to clarify direct regulatory mechanisms (e.g., protein-protein interactions).

Terpenoids are another class of essential metabolites involved in plant growth, defense, and signaling (Ninkuu et al., 2021). We found that LHT1-OE/KO reduces total terpene content in leaves, with LHT1-OE specifically decreasing diterpenoids and increasing triterpenoids. This metabolic shift is associated with downregulated *ACCT* (MVA pathway), *FPPS* (terpene precursor synthesis), and *TPS* (terpene diversification) genes, indicating that NtLHT1 regulates both MVA and MEP pathways. The differential effects of LHT1-OE and LHT1-KO (e.g., triterpenoid accumulation only in OE) suggest a dose-dependent role of NtLHT1 in terpene metabolism, which merits future exploration the intrinsic relationship between NtLHT1 and terpene metabolism.

As an amino acid transporter, NtLHT1’s ability to modulate hormone and secondary metabolism suggests it acts as a “metabolic node” linking primary (amino acid) and secondary (flavonoid, terpene) metabolism, as well as hormone biosynthesis. However, how the “metabolic node” works remain unclear and require further investigation.

In conclusion, this study demonstrates that NtLHT1 regulates tobacco leaf development and stress resistance by modulating CTK/IAA/GA hormone homeostasis, and repressing flavonoid and terpene biosynthesis via transcriptomic-metabolic reprogramming. These findings provide a molecular framework for understanding NtLHT1’s multifunctional roles and offer potential targets for improving agronomic traits (e.g., leaf size, stress tolerance) in tobacco and other crops through genetic manipulation of NtLHT1.

## Data Availability

All the data and material generated or analyzed during this study are included in this published article. The raw sequence data reported in this paper have been deposited in the Genome Sequence Archive in the National Genomics Data Center, China National Center for Bioinformation/Beijing Institute of Genomics, Chinese Academy of Sciences (GSA: CRA015607), which are publicly accessible at https://ngdc.cncb.ac.cn/gsa.

## Supplementary material

Supplementary Figure 1 Enrichment analysis of metabolite pathways in tobacco root, stem, and leaf in NtLHT1 overexpressing plants. **(A)** NtLHT1 overexpression significantly upregulated metabolites such as coumarins, phospholipids, nucleotides, and others in leaves. **(B)** NtLHT1 overexpression significantly downregulated metabolites such as diterpenes, flavonoids, ketones, and others in leaves. **(C)** NtLHT1 overexpression significantly downregulated metabolites such as diterpenes, flavonoids, organic acids, and others in stems. **(D)** NtLHT1 overexpression significantly downregulated metabolites such as amino acids, diterpenes, glycerides, sugars, sesquiterpenes, and others in tobacco roots.

Supplementary Figure 2 Enrichment analysis of upregulated and downregulated metabolites in tobacco root, stem, and leaf by NtLHT1 knockout. **(A)** NtLHT1 knockout significantly upregulated metabolites such as phospholipids, nucleotides, organic acids, and others in stems. **(B)** NtLHT1 knockout significantly upregulated metabolites such as amino acids, phospholipids, sugars, and others in roots. **(C)** NtLHT1 knockout significantly upregulated metabolites such as flavanols, phospholipids, pyridine, vitamins, and others in leaves. **(D)** LHT1 knockout significantly downregulated metabolites such as coumarins, diterpenes, and others in roots. **(E)** NtLHT1 knockout significantly downregulated metabolites such as diterpenes, ketones, phenols, and others in leaves. **(F)** NtLHT1 knockout significantly downregulated metabolites such as alkaloids, flavonoids, pinene, and others in stems.

Supplementary Figure 3 The 50 most significantly altered metabolites in tobacco root, stem, and leaf tissues in NtLHT1 overexpression and knockout. The x-axis represents different samples. The y-axis represents different categories of differentially regulated metabolites, and the color heatmap represents the relative content of metabolites in different samples. The results show significant changes in the content of peptides, flavonoid glycosides, amino acid derivatives, and other metabolites due to NtLHT1 overexpression and knockout in different tissues.

Supplementary Figure 4 Pathway enrichment analysis of upregulated and downregulated metabolites in the leaf and stem of LHT1-OE compared with LHT1-KO.

Supplementary Figure 5 Effects of NtLHT1 overexpression and knockout on tobacco leaf zeatin biosynthesis pathway. **(A)** Heatmap displaying the relative levels of selected zeatin related compounds in tobacco leaves of LHT1-WT, LHT1-KO and LHT1-OE plants. **(C)** Simplified schematic representation of zeatin biosynthesis pathway, showing major enzymatic reactions and intermediates.

Supplementary Figure 6 Analysis of *NtLHT1* gene and 6 DEGs expression level.

Supplementary Table 1 The primer sequences used in this study.

Supplementary Table 2 Results of metabolite quantification and annotation.

Supplementary Table 3 ANOVA analysis in root on LHT1- WT/OE/KO plants.

Supplementary Table 4 ANOVA analysis in stem on LHT1- WT/OE/KO plants.

Supplementary Table 5 ANOVA analysis in leaf on LHT1- WT/OE/KO plants.
