# Supplementary figures and images for: Integrative transcriptomic and metabolomic analyses provide insights into the effects of overexpression and knockout of NtLHT1 in different tissues
Source: Front Plant Sci. 2026 Feb 6;17:1663088. doi: 10.3389/fpls.2026.1663088 (PMC12920487; doi:10.3389/fpls.2026.1663088)

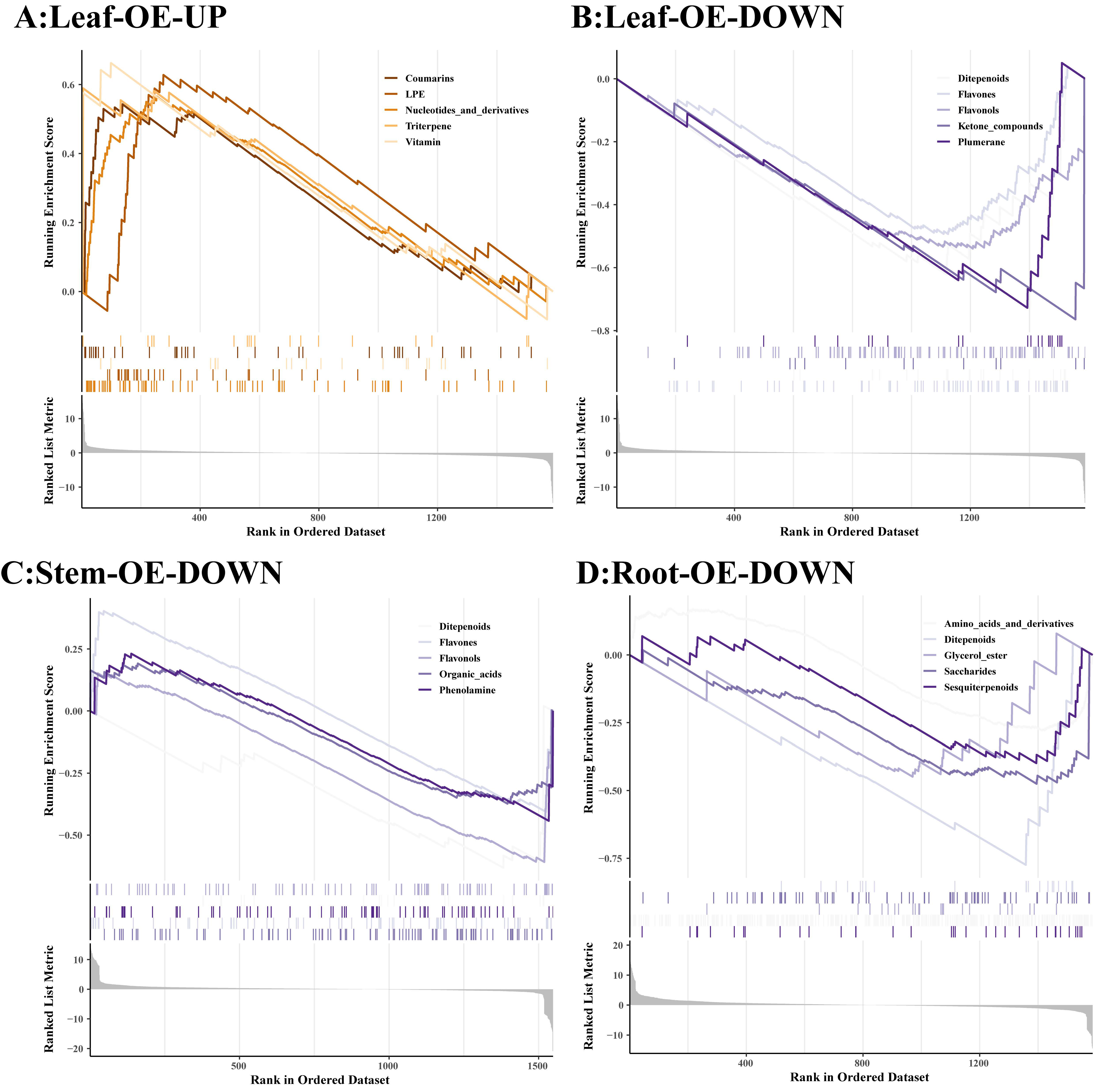

Supplement: Supplementary Figure 1 — Enrichment analysis of metabolite pathways in tobacco root, stem, and leaf in NtLHT1 overexpressing plants. (A) NtLHT1 overexpression significantly upregulated metabolites such as coumarins, phospholipids, nucleotides, and others in leaves. (B) NtLHT1 overexpression significantly downregulated metabolites such as diterpenes, flavonoids, ketones, and others in leaves. (C) NtLHT1 overexpression significantly downregulated metabolites such as diterpenes, flavonoids, organic acids, and others in stems. (D) NtLHT1 overexpression significantly downregulated metabolites such as amino acids, diterpenes, glycerides, sugars, sesquiterpenes, and others in tobacco roots. [file Image1.png]

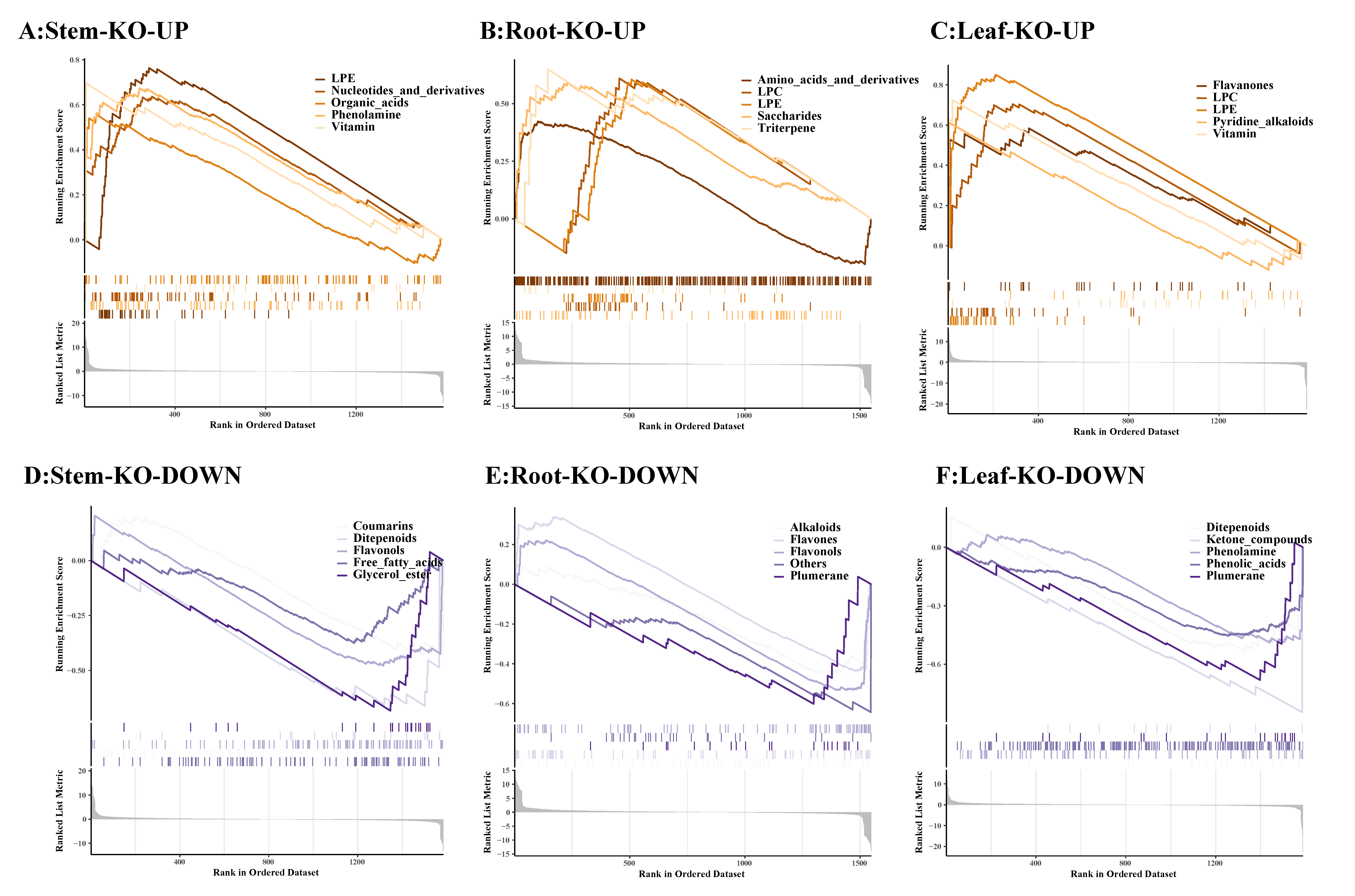

Supplement: Supplementary Figure 2 — Enrichment analysis of upregulated and downregulated metabolites in tobacco root, stem, and leaf by NtLHT1 knockout. (A) NtLHT1 knockout significantly upregulated metabolites such as phospholipids, nucleotides, organic acids, and others in stems. (B) NtLHT1 knockout significantly upregulated metabolites such as amino acids, phospholipids, sugars, and others in roots. (C) NtLHT1 knockout significantly upregulated metabolites such as flavanols, phospholipids, pyridine, vitamins, and others in leaves. (D) LHT1 knockout significantly downregulated metabolites such as coumarins, diterpenes, and others in roots. (E) NtLHT1 knockout significantly downregulated metabolites such as diterpenes, ketones, phenols, and others in leaves. (F) NtLHT1 knockout significantly downregulated metabolites such as alkaloids, flavonoids, pinene, and others in stems. [file Image2.tiff]

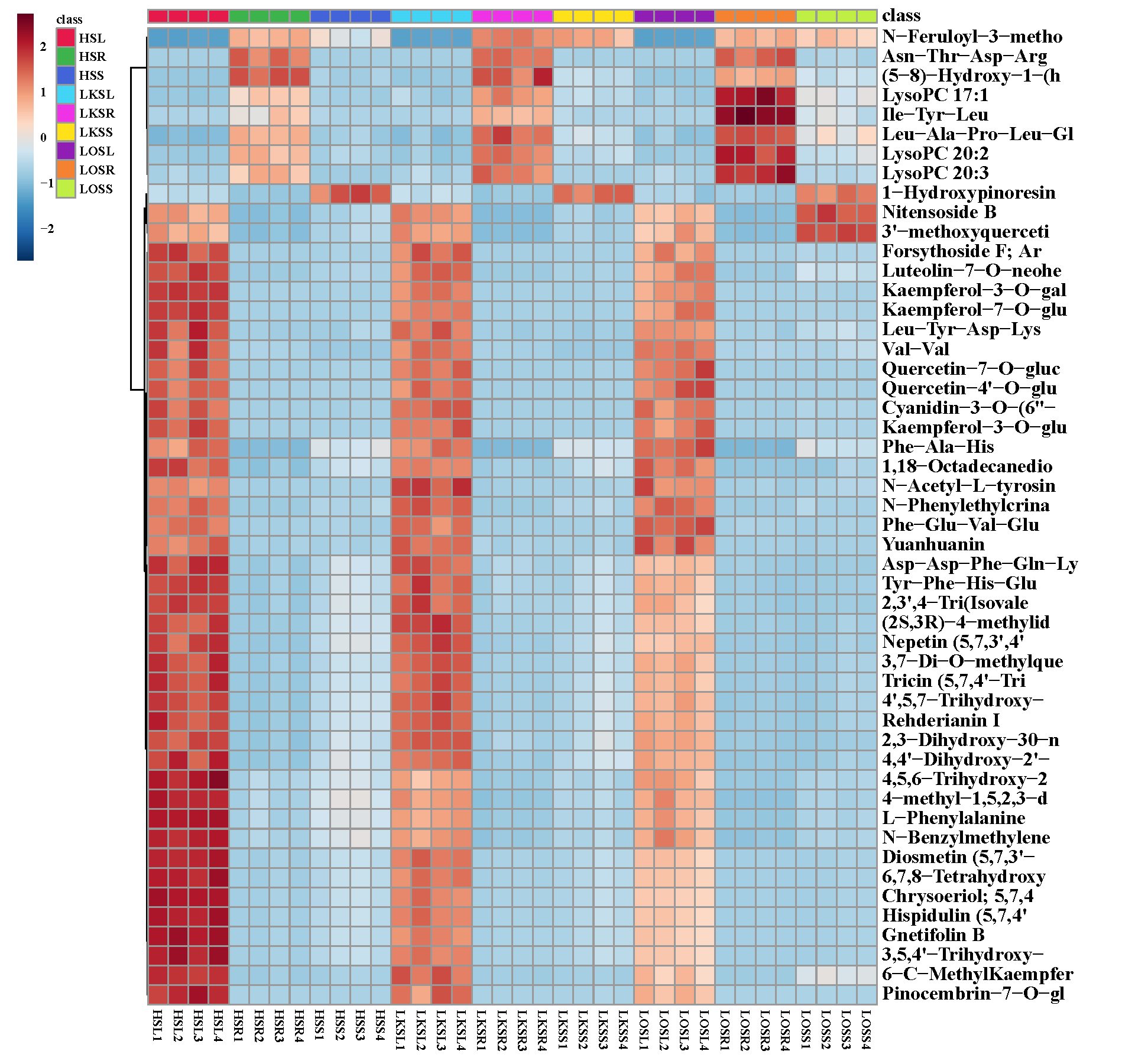

Supplement: Supplementary Figure 3 — The 50 most significantly altered metabolites in tobacco root, stem, and leaf tissues in NtLHT1 overexpression and knockout. The x-axis represents different samples. The y-axis represents different categories of differentially regulated metabolites, and the color heatmap represents the relative content of metabolites in different samples. The results show significant changes in the content of peptides, flavonoid glycosides, amino acid derivatives, and other metabolites due to NtLHT1 overexpression and knockout in different tissues. [file Image3.tiff]

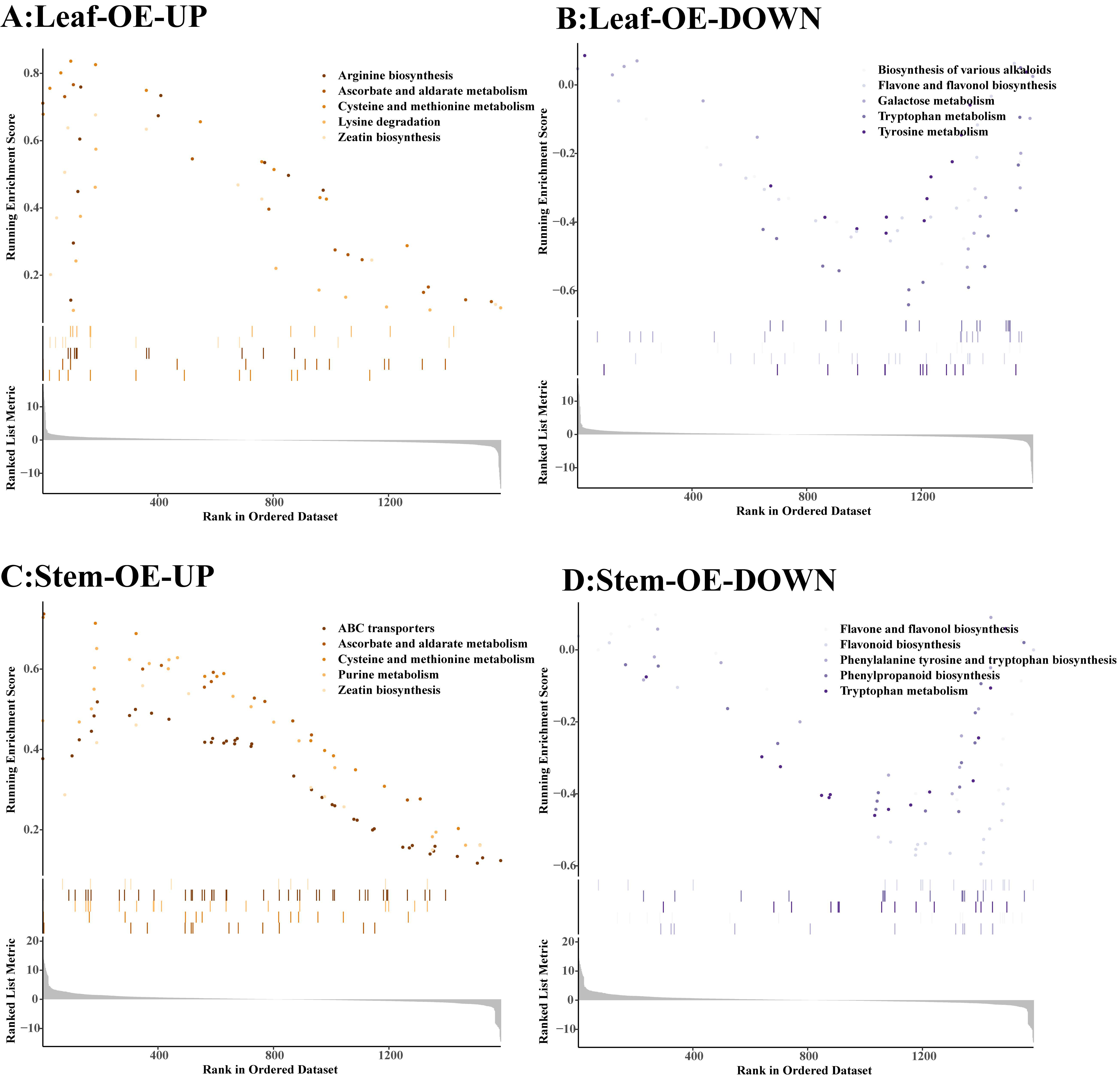

Supplement: Supplementary Figure 4 — Pathway enrichment analysis of upregulated and downregulated metabolites in the leaf and stem of LHT1-OE compared with LHT1-KO. [file Image4.png]

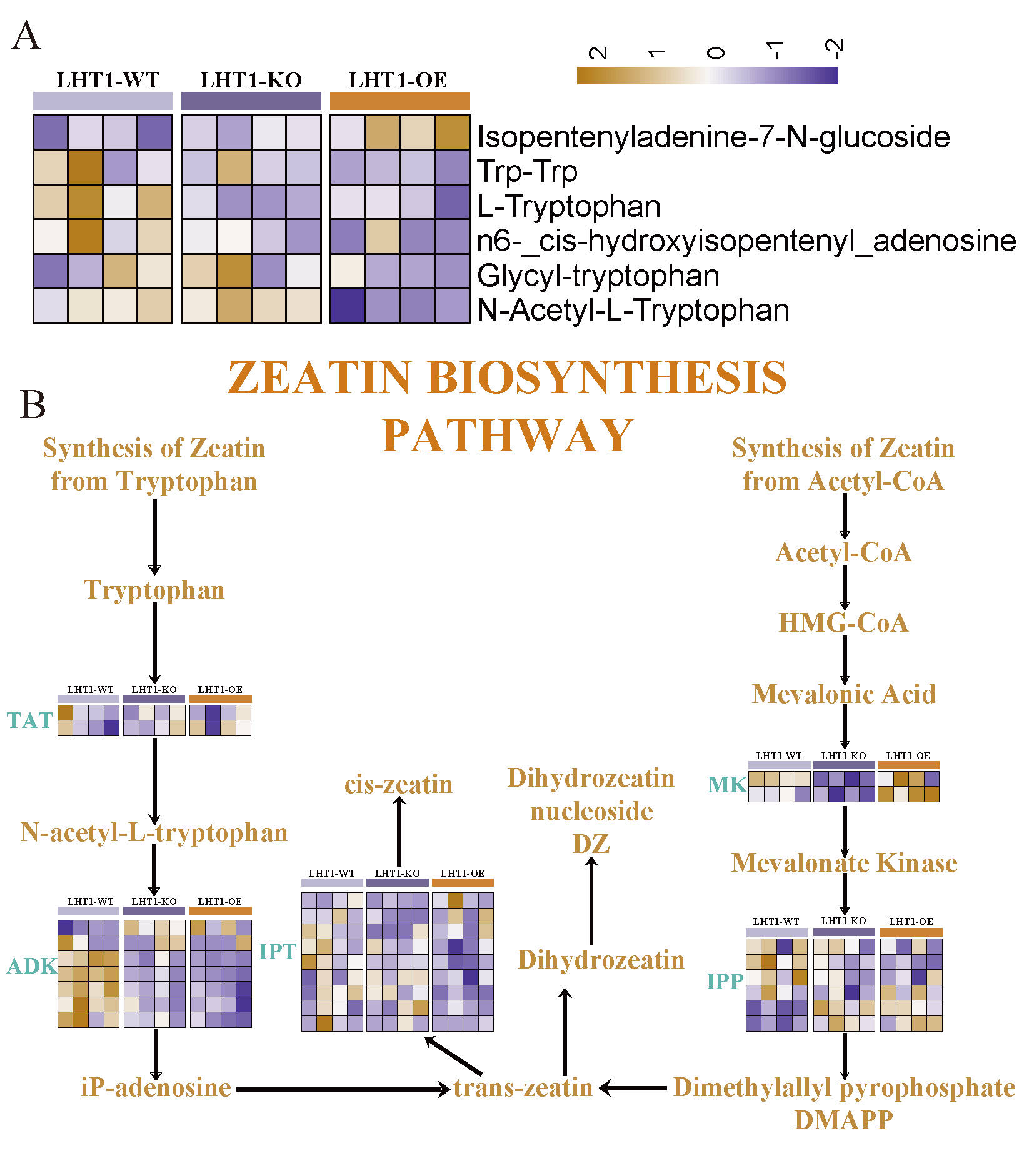

Supplement: Supplementary Figure 5 — Effects of NtLHT1 overexpression and knockout on tobacco leaf zeatin biosynthesis pathway. (A) Heatmap displaying the relative levels of selected zeatin related compounds in tobacco leaves of LHT1-WT, LHT1-KO and LHT1-OE plants. (C) Simplified schematic representation of zeatin biosynthesis pathway, showing major enzymatic reactions and intermediates. [file Image5.tif]

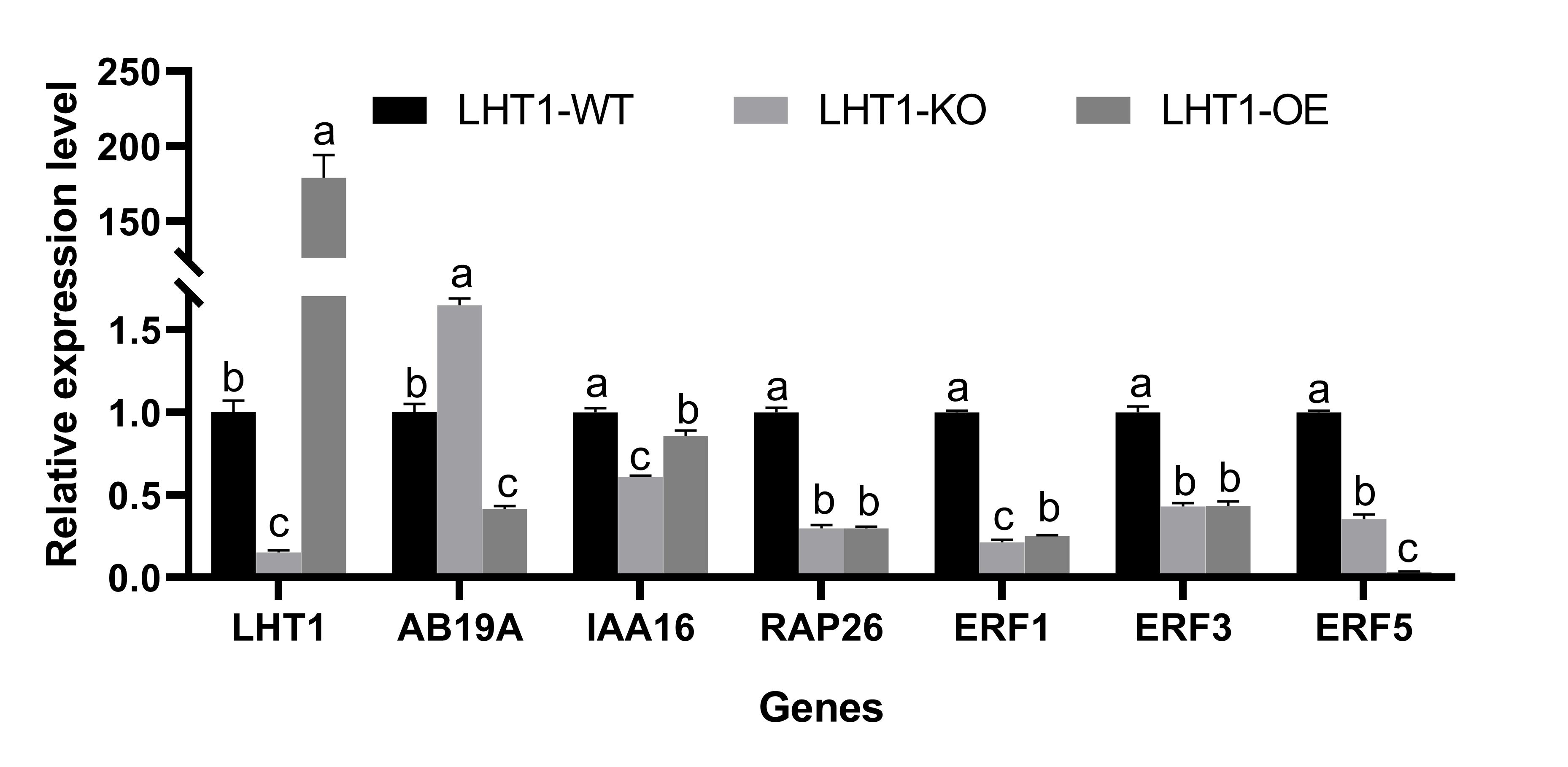

Supplement: Supplementary Figure 6 — Analysis of NtLHT1 gene and 6 DEGs expression level. [file Image6.jpeg]
